# Omega Phase Formation in Ti–3wt.%Nb Alloy Induced by High-Pressure Torsion

**DOI:** 10.3390/ma14092262

**Published:** 2021-04-27

**Authors:** Anna Korneva, Boris Straumal, Askar Kilmametov, Alena Gornakova, Anna Wierzbicka-Miernik, Lidia Lityńska-Dobrzyńska, Robert Chulist, Łukasz Gondek, Grzegorz Cios, Paweł Zięba

**Affiliations:** 1 Institute of Metallurgy and Materials Science, Polish Academy of Sciences, 30-059 Krakow, Poland; a.wierzbicka@imim.pl (A.W.-M.); l.litynska@imim.pl (L.L.-D.); r.chulist@imim.pl (R.C.); p.zieba@imim.pl (P.Z.); 2 Karlsruhe Institute of Technology (KIT), Institute of Nanotechnology, 76344 Eggenstein-Leopoldshafen, Germany; straumal@issp.ac.ru; 3 Institute of Solid State Physics, Russian Academy of Sciences, 142432 Chernogolovka, Russia; alenahas@issp.ac.ru; 4 Scientific Center in Chernogolovka, Russian Academy of Sciences, 142432 Chernogolovka, Russia; askar.kilmametov@kit.edu; 5 Faculty of Physics and Applied Computer Science, AGH University of Science and Technology, Mickiewicza 30, 30-059 Kraków, Poland; lgondek@agh.edu.pl; 6 Academic Centre of Materials and Nanotechnology, AGH University of Science and Technology, Mickiewicza 30, 30-059 Kraków, Poland; grzegorz.cios@agh.edu.pl

**Keywords:** high-pressure torsion, in situ high-temperature X-ray diffraction, Ti alloys, phase transformations, scanning electron microscopy, transmission electron microscopy, differential scanning calorimetry

## Abstract

It is well known that severe plastic deformation not only leads to strong grain refinement and material strengthening but also can drive phase transformations. A study of the fundamentals of α → ω phase transformations induced by high-pressure torsion (HPT) in Ti–Nb-based alloys is presented in the current work. Before HPT, a Ti–3wt.%Nb alloy was annealed at two different temperatures in order to obtain the α-phase state with different amounts of niobium. X-ray diffraction analysis, scanning electron microscopy (SEM) and transmission electron microscopy (TEM) were applied for the characterisation of phase transitions and evolution of the microstructure. A small amount of the β-phase was found in the initial states, which completely transformed into the ω-phase during the HPT process. During HPT, strong grain refinement in the α-phase took place, as did partial transformation of the α- into the ω-phase. Therefore, two kinds of ω-phase, each with different chemical composition, were obtained after HPT. The first one was formed from the β-phase, enriched in Nb, and the second one from the α-phase. It was also found that the transformation of the α-phase into the ω-phase depended on the Nb concentration in the α-Ti phase. The less Nb there was in the α-phase, the more of the α-phase was transformed into the ω-phase.

## 1. Introduction

Ti–Nb alloys are characterised by a variety of metallurgical engineering routes that allow a varied range of microstructural configurations and physical properties to be obtained by careful selection of chemical composition, heat treatment and mechanical processing [1]. Ti–Nb-based alloys are versatile candidates for biomedical applications because they exhibit excellent corrosion resistance, with a low modulus of elasticity close to that of human bone, as well as good ductility, allowing medical devices to be processed with precise and versatile geometries [2,3]. However, it is expected that Ti–Nb-based alloys may possess lower strength in comparison with widely applied biomaterials such as Ti–5Al–2.5Fe, Ti–6Al–4V and Ti–6Al–7Nb alloys. These conventional biomaterials are commonly used despite their quite high Young’s moduli and rather limited biocompatibility [4,5]. It is well known that mechanical properties, especially strength, can be significantly improved by applying severe plastic deformation (SPD) as a result of the increased density of crystal lattice defects and strong grain refinement reaching ultrafine size or even the nanometer scale [6,7]. In this context, it became possible to strengthen ternary and quaternary Ti-based alloys with niobium as a β-stabilizer, using SPD techniques such as equal channel angular pressing (ECAP) or high-pressure torsion (HPT) [8,9,10,11]. HPT can also drive several phase transitions, such as precipitation or dissolution of a second phase particles [12,13,14], formation or decomposition of an amorphous phase [15,16] and transformations between allotropic variants of the same element [17]. SPD is quite effective for stimulating allotropic transformations, for example, in titanium, zirconium or hafnium alloys which possess a high-temperature body-centred cubic Im3m β-phase, a low-temperature hexagonal close-packed crystal structure P63/mmc α-phase and a high-pressure hexagonal P6/mmm ω-phase [1,18,19]. In Ti-based alloys, the ω-phase can be obtained either as a result of slow quenching from the β-phase (further referred to as the ω_athermal_-phase), or during isothermal aging (the ω_isothermal_-phase [20]). The ω_athermal_-phase forms by a displacive diffusionless mechanism, while the ω_isothermal_-phase is stabilised by diffusion [21]. When high cooling rates are applied to alloys in the β-phase, two other metastable phases, depending on the alloy composition, can be formed by a diffusionless mechanism, competing with the ω-phase. They are α′ martensite, with hexagonal closely packed structure, and α″ martensite, with orthorhombic structure [2,22,23].

It was observed that, in Ti alloys, a high-pressure ω-phase can form during SPD from the β-phase, as well as from the α-phase under hydrostatic pressure from 2 to 12 GPa [24,25,26]. The α → ω and β → ω phase transformations induced by SPD are typical (diffusionless) martensitic transformations. The alloying of Ti with β-stabilizers (like Co, Ni, Fe, Nb, Mo) and shear strain promote the formation of the ω-phase. Recently, much attention has been paid to the formation of the high-pressure ω-phase from the α-phase of titanium of commercial purity [27,28], from the β-phase or from α′ martensite of Ti–Fe-based alloys during HPT [29,30]. However, the fundamentals of the SPD-induced α → ω phase transformation as well as the subsequent thermal stability of the ω-phase in Ti–Nb alloys are poorly studied. Therefore, the main goal of this work was to investigate the influence of the addition of an alloying component (Nb) on the mechanisms of HPT-driven α → ω phase transformation in a Ti–3 wt.% Nb alloy and to study the thermal stability of the ω-phase as well as the microhardness change under the influence of the ω-phase.

## 2. Material and Methods

A Ti–3wt.% Nb alloy in the form of 10 mm diameter cylindrical ingots was manufactured from pure titanium (99.98%) and niobium (99.99%) by melting in a pure argon atmosphere in an induction furnace. The ingots were then cut by spark erosion into 0.7 mm thick discs. These discs were then individually sealed in silica ampoules at a residual pressure of 4 × 10^−4^ Pa and annealed at two different temperatures (450 °C for 720 h and 600 °C for 888 h). After annealing, the α-phase contained different amounts of niobium. The long-term annealing took place in order to produce an α-solid solution with equilibrium Nb concentration. After annealing, the silica ampoules with samples were quenched in cold water. The annealed samples were subjected to HPT. HPT was performed in a Bridgman anvil type unit (manufactured by W. Klement GmbH, Lang, Austria) at 25 °C, 7 GPa, with five anvil rotations and a rotation rate of 1 rpm. The strain rate at a distance of half the radius of the deformed disc reached 4.3 10^−1^ s^−1^ [31]. The thickness of the samples after HPT was 0.35 mm. Transmission electron microscopy (TEM) studies were carried out using a TECNAI G2 FEG super TWIN (200 kV) (FEI, Hillsborough, OR, USA) with an energy-dispersive X-ray (EDS) spectrometer produced by EDAX (AMETEK, Inc., Berwyn, PA, USA). The thin foils for TEM observation were prepared by a twin-jet polishing technique using a D2 electrolyte manufactured by Struers Inc. (Cleveland, OH, USA). The thin samples of deformed material were produced in order to observe the interface between the second phase and the α-matrix. This was done using the focused ion beam (FIB) technique with FIB Quanta 3 D, TECNAI FEG microscopy (30 kV) (FEI, Hillsborough, OR, USA). The X-ray diffraction (XRD) studies were carried out with Cu–Kα radiation with the aid of a Siemens D-500 X-ray diffractometer (Malvern Panalytical, Malvern WR14 1XZ, UK). The lattice parameter values were calculated using Rietveld-like whole profile refinement by the Fityk programme [32]. The measurement error of lattice parameters was ±0.00001 nm for the well-resolvable coarse-grained states and ±0.0001 nm for the deformed states. Prior inspection of the obtained material was also performed using an FEI E-SEM XL30 (FEI, Hillsborough, OR, USA) scanning electron microscope (SEM) equipped with an EDAX Genesis EDS spectrometer (FEI, Hillsborough, OR, USA). The composition contrast between different phases became visible in the SEM images by using the backscattered electron signal in BSE mode. All the microstructural studies were carried out using specimens cut out at a half-radius distance from the centre of HPT discs to avoid microstructure heterogeneity between the centre and the periphery of the deformed samples. The stereological analysis was performed in an automatic regime basing on all the pixels of each photo, by means of the ImageJ by Wayne Rasband 1.45 s software, National Institutes of Health (Bethesda, MD, USA) [33]. Series of 5 independent SEM images were taken into account to provide full information on the percentage fraction of structural components. The regions of different phases were isolated at the sample surface, and then the total relative area was determined in all the images. Next, only the selected phase field was separated, and the relative area of the given phase was measured. The in situ XRD investigations were done using a Panalytical Empyrean diffractometer (Malvern Panalytical, Malvern WR14 1XZ, UK) (Cu-*Kα* radiation) equipped with an Anton Paar HTK 1200 high-temperature chamber (Malvern Panalytical, Malvern WR14 1XZ, UK). The samples for the in situ XRD investigations were placed on an Al_2_O_3_ sample holder and then put into the diffractometer sample chamber. The chamber was then evacuated, flushed and filled with high-purity Ar gas (6N). The thermal displacement of the samples during measurements was subtracted by the appropriate movement of the sample chamber. Heating took place at a rate of up to 850 °C, at 5 °C/min. The diffraction patterns were collected at steps of 20 °C. We chose the 2θ range between 30° and 80°, and the step size was 0.01667°. The acquisition was preceded by 10 min of temperature stabilisation, and the acquisition time per single pattern was 25 min. The collected data were refined using Rietveld-type Fityk software [32]. Differential scanning calorimetry (DSC) was carried out using 404 F1 Pegasus, Netzsch equipment (Mettler Toledo, Columbus, OH, USA). The samples were put into Al_2_O_3_ crucibles in an argon atmosphere and heated up at a rate of 20 °C/min up to 900 °C. For hardness measurements we used an AGILENT G200 nanoindenter (Keysight Technologies, Santa Rosa, CA, USA) with an XP head at a load of 96 mN. We measured the hardness with steps of 250 µm from the centre to the edge of the sample along the radius of the deformed discs. For each radius value, we made 10 hardness measurements and calculated the average.

## 3. Results and Discussion

**Figure 1:**
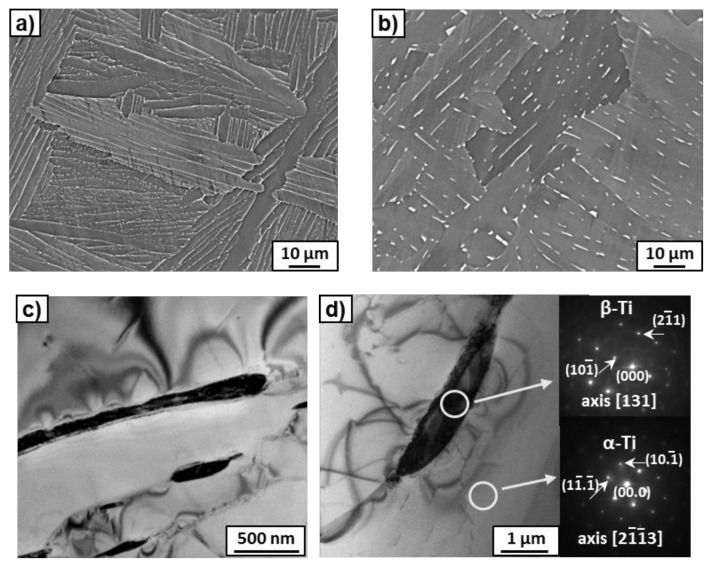
(**a**,**b**) SEM and (**c**,**d**) TEM micrographs of the Ti–3wt.% Nb alloy annealed at 450 °C (**a**,**c**) and 600 °C (**b**,**d**). Bright-field images (**c**,**d**) with SAED patterns indicated the β- and α-phases. The white circles marked in the image (**d**) show the areas from which SAED patterns were obtained.

Microstructure observations of the Ti–3wt.% Nb alloy after annealing at 450 °C and 600 °C by SEM (Figure 1a,b) showed the presence of a small amount of β-phase precipitates, uniformly distributed in the α-matrix. The β-phase grains were enriched in niobium and were bright in comparison with the dark α-matrix in the micrographs taken in the BSE mode. In Figure 1a, they look like thin layers of bright contrast located at the boundaries between grains of the dark α-phase. Using precise phase separation at SEM images, the volume fraction of β-phase was evaluated to be about 4 and 8% in the samples annealed at 450 °C and 600 °C, respectively. The study of selected area, electron diffraction patterns (SAED), obtained by TEM, also made it possible to identify precipitates of the second phase as a β-phase surrounded by the α-matrix (Figure 1c). Measurement of the chemical composition by means of EDS during the TEM study showed an increase of niobium content in the α-matrix from 2.2 to 3.0 wt.%, with an increase of the annealing temperature from 450 to 600 °C. According to Ref. [18], the maximum solubility of Nb in α-Ti is reached between 600 and 650 °C and about 2.0–2.5 at.% Nb, which corresponds to 3.8–4.7 wt.% Nb. Therefore, the results of our experiments correspond to those published in the literature.

**Figure 2:**
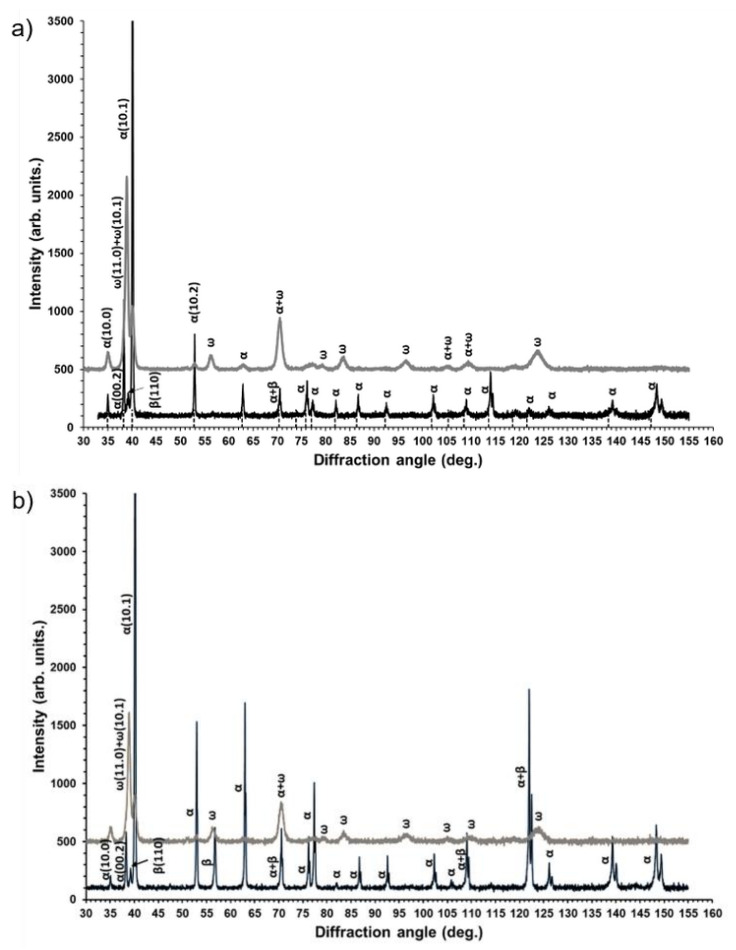
XRD patterns of the Ti–3wt.% Nb alloy before (black curves) and after (gray curves) HPT deformation for the samples pre-annealed at 450 °C (**a**) and 600 °C (**b**).

Figure 2 shows the presence of the α- and β-phases at XRD patterns of the samples in the initial state. Substantial volume fraction of the α-phase took place for both initial states and consisted of approximately 90%. Increasing the annealing temperature from 450 to 600 °C led to a slightly elevated volume fraction of the β-phase in accordance with the Ti–Nb phase diagram. HPT resulted in a considerable broadening of the XRD peaks, which is typically due to strong grain refinement. At the same time, the vast majority (70–80%) of the ω-Ti phase after HPT was observed in both cases (Figure 2). It is clear that the peaks of the β-phase disappeared, but a certain amount of the α-Ti phase remained after HPT. These facts obviously indicated α → ω phase transformation under HPT conditions, although the formation of the ω-phase needs to be considered in more detail. Recently, it was found that even a 0.1 rotation of HPT deformation of a Ti–4wt.%Fe alloy in the β-state led to the formation of 90% of the ω-phase [34]. This indicates that the high-pressure ω-phase forms more easily from the β-phase during HPT. By analysis of the α → ω phase transformation, a specific crystallographic relationship was established between the grains of α- and ω-phases [34,35]. It assumes that the shear strain along the (00.1) planes promotes α- to ω- transformation [35]. For both initial states of the Ti–Nb alloy, Figure 2 shows redistribution of the XRD peak intensities, which is typical for α-to-ω phase transformation under HPT conditions. This means that the mechanism of the α-to-ω transitions for the Ti–Nb alloy in our case was similar to that previously observed for pure Ti [24] and Ti-based alloys [34,35].

The lattice parameters of the observed phases were also calculated using the XRD patterns (Table 1). The lattice parameters of the α-phase in the sample annealed at 450 °C were close to those of pure titanium [36]. An increase of the annealing temperature up to 600 °C resulted in a decrease of the lattice parameters of the α-phase and, as a consequence, to a decrease of cell volume, which can be associated with the increase of niobium content in the cell (Table 2). These measurements are in a good agreement with a marked decrease in the values of the lattice parameters that took place in the Ti-based alloys doped with Fe, Cr, Ni and Co in the range of less than 1 wt.% [37]. After deformation, the cell volume of the α-phase in the alloys, preliminarily annealed at 600 °C, was lower than that in the alloys annealed at 450 °C. The increase of the cell volume may indicate a tendency towards HPT-induced niobium depletion of the α-matrix solid solution. This behaviour after HPT is similar to the recently observed phenomenon in the Ti–Fe alloys, which was called “purification” (or solute lean) of the α-phase [30]. It is likely that niobium atoms migrate during HPT from the “old” α-phase into the “new” ω-phase. As is well known, the solubility of Fe in the α-Ti phase is much lower than in the ω-Ti phase [34]. Based on this, it can be supposed that the solubility of niobium in the α-Ti phase is also much lower than in the ω-Ti phase.

**Figure 3:**
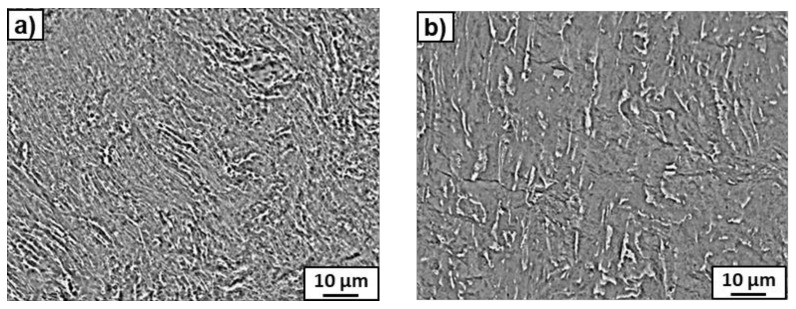
SEM images of the microstructure of HPT-deformed samples of the Ti–3wt.%Nb alloy pre-annealed at 450 °C (**a**) and 600 °C (**b**).

**Figure 4:**
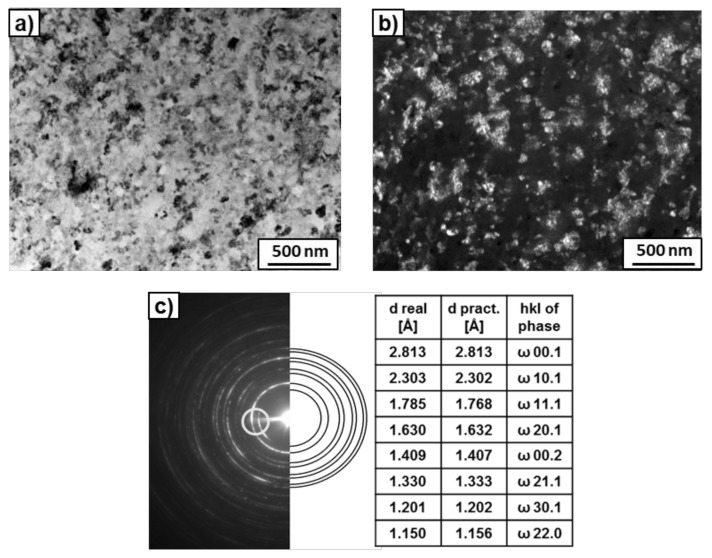
TEM micrographs of the Ti–3wt.% Nb alloy after HPT deformation and pre-annealing at 600 °C. Bright field (**a**), dark field (**b**) images, as well as SAED pattern (**c**). The ring shows the position of the objective aperture for obtaining the dark-field image. The solutions are given in the table (**c**).

**Figure 5:**
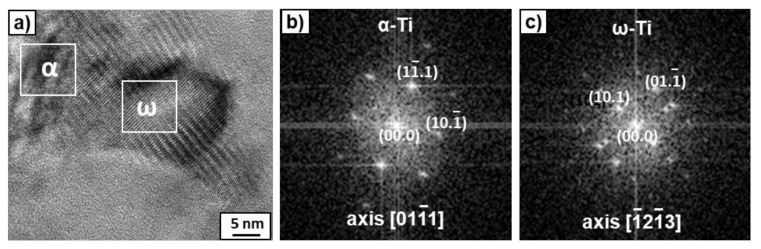
High-resolution TEM image (**a**) of the microstructure of the HPT-deformed Ti–3wt.% Nb alloy, pre-annealed at 600 °C with Fast Fourier Transforms (FFT) obtained from the marked squares for the α-phase (**b**) and ω-phase (**c**).

After the HPT process, microstructure refinement and deformation of the bright phase were clearly visible in the SEM micrographs (Figure 3). This phase can belong to the suspected prior beta grains. However, if the β-phase disappeared completely, to which phase does the bright precipitation belong? The answer to this question was obtained by studying the microstructure using TEM. Figure 4 presents the microstructure of the deformed sample previously annealed at 600 °C. It shows a strong grain refinement of the microstructure down to nm sizes. The SAED pattern (that was taken from the whole area, presented in Figure 4a) showed only reflexes from the ω phase in this sample, as well as a small amount of the α phase in the case of the sample previously annealed at 450 °C. It is possible that, due to the small amount of α-phase in the samples, only its single reflections could be seen in the SAED patterns and, therefore, the software used for the calculation of lattice spacing in the electron diffraction patterns [38] did not recognize them. However, the presence of the α-phase was detected by means of high-resolution TEM studies (Figure 5). High-resolution TEM studies were performed on the same sample, as shown in Figure 4. The HREM observation made the separate grains visible. The columns of crystal atoms were visible in the RHEM image if they were parallel to the electron beam. In Figure 5, the atoms columns of two grains are visible (the light gray colour corresponds to the grains for which the columns were not parallel to the electron beam). The measured distances d_hkl_ and the angle between them in the Fast Fourier Transforms of the selected grains corresponded to the α- and ω- phases. This implies that the α phase was also present in this sample.

**Figure 6:**
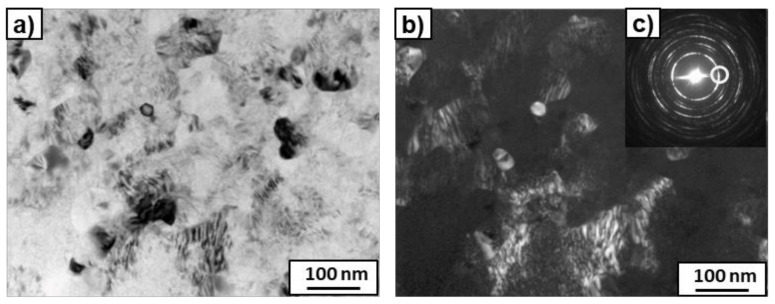
TEM micrographs of the Ti–3wt.% Nb alloy after HPT deformation and pre-annealing at 600 °C. Bright field (**a**), dark field (**b**) images and SAED pattern (**c**). The ring shows the position of the objective aperture for obtaining the dark-field image.

**Figure 7:**
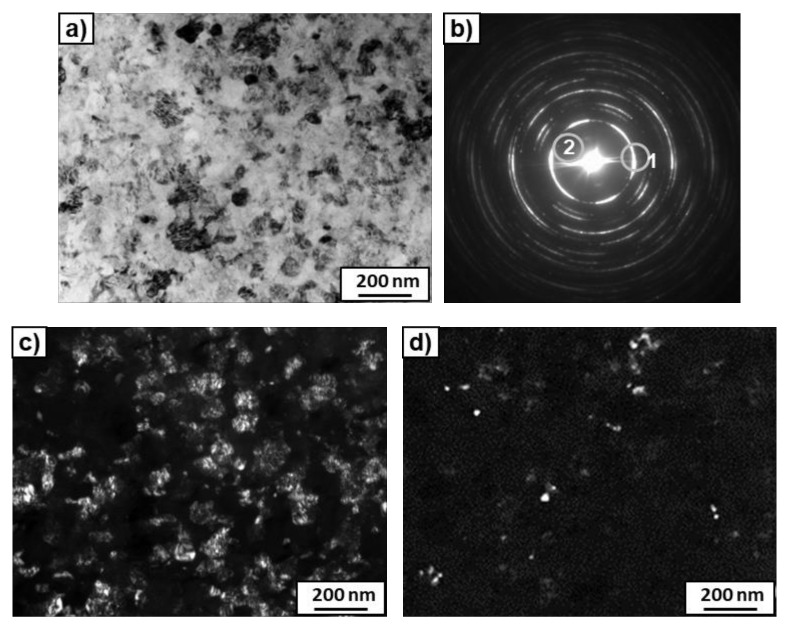
TEM micrographs of the Ti–3wt.% Nb alloy after HPT deformation and pre-annealing at 600 °C. Bright field (**a**), dark field (**c**,**d**) images and SAED pattern (**b**). The grey rings show the positions of the objective aperture for obtaining the dark-field images (rings 1 and 2 correspond to (**c**) and (**d**) dark field images, respectively).

High-magnification TEM observations of the microstructure (Figure 6) showed small grains of rounded shape and large grains of irregular shape with distinctive streaky contrast. The morphology of the large grains was similar to the ω-phase [39], while the small grains belong to the α-phase. This was confirmed by electron diffraction analysis of the selected area, presented in Figure 7b. Ring 1 marked in SEAD shows mainly reflexes (10.1) of the ω-phase and corresponds to the dark field in Figure 7c, where large grains of irregular shape with distinctive streaky contrast are clearly visible. Ring 2 contains individual reflexes, the interatomic distance of which corresponds to the reflexes (10.0) of the α-phase. Ring 2 is responsible for the dark field in Figure 7d, where a few small grains of rounded shape are visible.

**Figure 8:**
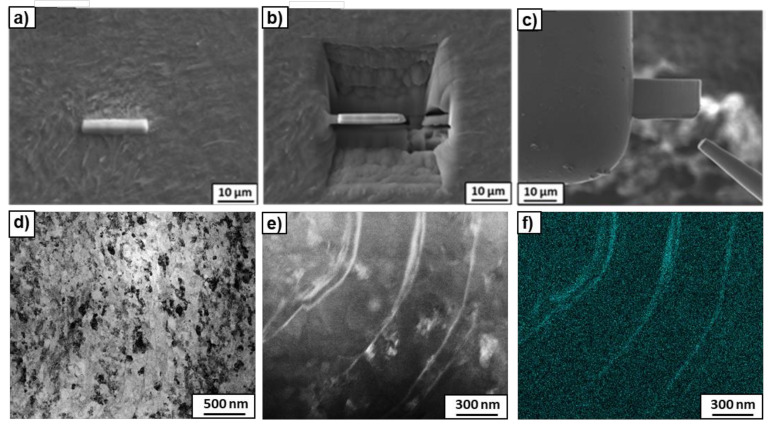
Some stages of thin foil preparation using the FIB method (**a**–**c**). Bright-field image (**d**), HAADF-STEM image (**e**) of the HPT-deformed Ti–3wt.% Nb alloy, pre-annealed at 600 °C and mapping of Nb element (**f**).

The preparation of thin foils using the FIB method was performed in order to find what happened to the β- phase, i.e., to understand the nature of the noted bright phase clearly visible in the SEM micrographs (Figure 3) after the HPT process. During the preparation of the thin foil, a strip of platinum was applied to the sample surface across three elongated precipitates of the bright contrast (Figure 8a). The high-angle annular dark-field (HAADF) observations of the thin foil showed the presence of thin elongated precipitates of rounded shape, enriched in Nb up to 14.7 wt.% (Figure 8e,f). These precipitates originated from thin plates of the former β-phase, which changed their shape during torsion, to the extent that some of them were even bent (see Figure 3b). It should be noted that the chemical composition of 14.7 wt.% (measured by means of EDS during TEM study) was close to the β-phase in the initial state (14.5 wt.%Nb). Since no reflections of the β-phase were observed after HPT and the β → ω transformation is a martensitic process proceeding without changing the composition, it can be concluded that the β-phase completely transformed into the ω-phase. Therefore, the bright-phase precipitates observed after HPT in Figure 8e,f belonged to the ω-phase enriched in niobium, which was transformed from the β-phase. The amount of this kind of ω-phase corresponded to the amount of the β-phase in the initial state, i.e., from a few to approximately 8% for the samples pre-annealed at 450 °C and 600 °C, respectively. However, XRD analysis showed that the total amount of the ω-phase for the examined samples reached about 84 and 79%, respectively (Table 2). This means that the remaining amount of the ω-phase appeared from the partial transformation of the α-phase. Therefore, two kinds of ω-phase were observed, the first from the β-phase enriched in niobium, and the second from the almost niobium-free α-phase. Moreover, it turned out that the less Nb in the α-phase, the more the α-phase was transformed into the ω-phase (Table 2). For example, about 80% of the α-phase was transformed into the ω-phase in the sample pre-annealed at 450 °C, while in the sample pre-annealed at 600 °C, it was only 71%. Similar results were also obtained in a Ti–4wt.% Co alloy subjected to HPT under the same conditions [40], where it was observed that the α → ω phase transition during HPT was controlled by the cobalt concentration in the initial α-phase and microstructure peculiarities. The higher amount of ω-phase after HPT was observed at lower Co concentration and with smaller grains in the α- phase [40].

It should be noted that HAADF observation of the thin foil prepared from the deformed samples using the conventional twin-jet polishing technique was also performed. However, mapping of Nb in this case showed no difference in the structure in terms of niobium content. Possibly, the investigated foil regions mainly contained the ω-phase transformed from the α-phase and some α-deformed grains, whose chemical compositions did not differ.

**Figure 9:**
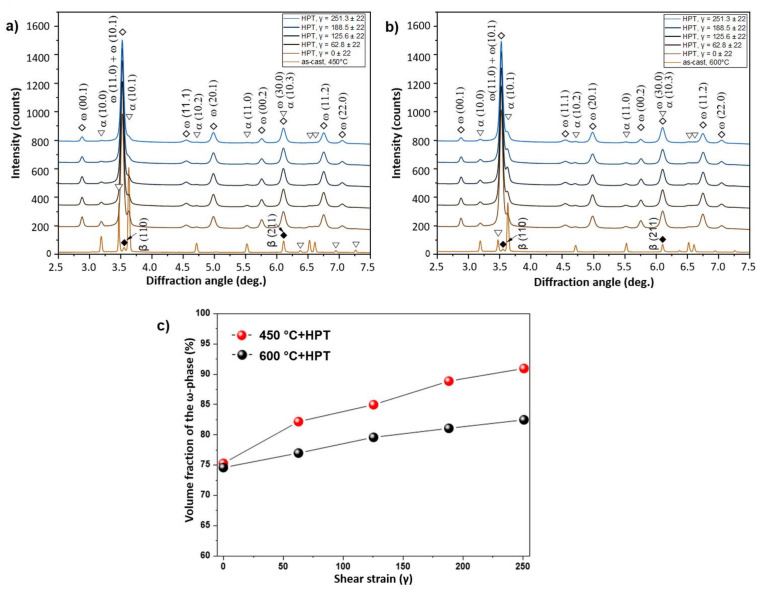
Synchrotron XRD curves of the deformed Ti–3wt.% Nb alloy, pre-annealed at 450 °C (**a**) and 600 °C (**b**). Volume fraction of the ω-phase as a function of the shear strain (**c**).

Synchrotron X-ray analysis was performed along the radius of the HPT-deformed samples. Based on the obtained X-ray curves (Figure 9a,b), the amount of the ω-phase was calculated as a function of the shear strain (Figure 9c). The higher the shear strain, the greater the amount of the deformation-induced ω-phase.

**Figure 10:**
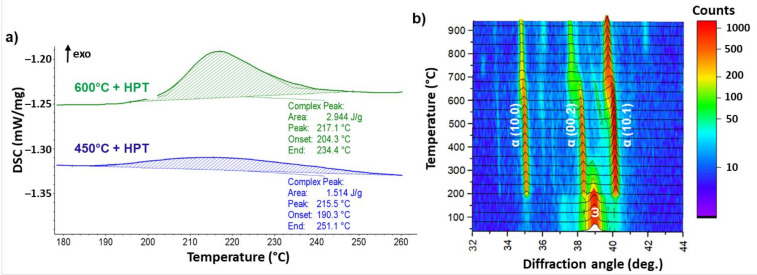
Enlarged part of the DSC heating curves of the deformed Ti–3wt.% Nb alloy pre-annealed at 450 and 600 °C (**a**). The XRD in situ heating map of the deformed Ti–3wt.% Nb alloy, pre-annealed at 600 °C (**b**). At the given 2θ angle interval, the ω-phase is presented by (11.0 + 10.1) a doublet of peaks.

DSC and in situ XRD at high-temperature were used to investigate the thermal stability of metastable ω-phase. Part of the DSC heating curves of the deformed Ti–3wt.% Nb alloy is presented in Figure 10a. The exothermic peaks in the temperature range of 190–250 °C and 204–234 °C, respectively, are clear in the DSC curves for the samples pre-annealed at 450 and 600 °C (Figure 10a). They could be due to the ω → α reverse transformation. The ω-phase is the metastable one, therefore the ω → α transition is exothermic. The exothermic nature of the reverse ω → α transformation was also observed in Ti–Fe- and Ti–Co-based alloys subjected to HPT deformation [29,40]. It can be seen in Figure 10a that the amount of the ω-phase can be estimated by calculating the areas under the respective exothermic peaks. The maximal exothermic peak was observed in the sample pre-annealed at 600 °C. In this sample, the amount of the ω-phase was also maximal.

The study of ω-phase thermal stability by in situ XRD measurements presented good correlation with the data obtained by the DSC method. An in situ XRD map of the HPT-deformed sample pre-annealed at 600 °C is presented in Figure 10b as an example. After deformation, the (11.0) + (10.1) peak of the ω-phase as well as the (10.0) and (10.1) peaks of the α-phase are clearly visible in the range of 2θ angles between 33° and 42° on the standard XRD curves (Figure 2). However, in the in situ XRD patterns before heating, only the (11.0) + (10.1) peak of the ω-phase can be observed. The absence of the α-phase peaks in this range of 2θ angles in the in situ XRD map is related to the different duration of XRD measurements. In comparison to the standard XRD method, there was not enough time to register many X-ray counts using the in situ method. The XRD in situ map shows that, after heating the deformed samples up to 200–250 °C, the ω-phase fully disappeared. The (10.0), (00.2) and (10.1) α-phase peaks appeared instead. Therefore, decomposition of the ω-phase into the α-phase was observed. The ω-phase in our samples was enriched in niobium (similar to the Fe-rich ω-phase in Ti–Fe alloys [24,30]). This suggests that the α-phase, which appeared after the decomposition of the ω-phase, conserved the Nb atoms and became enriched in niobium. At the highest heating temperatures, the α-phase peaks shifted slightly towards the low diffraction angles. This shift of the α-phase peaks can be associated with the appearance of the new α-phase with lower cobalt content, as well as with the increase of the lattice parameters due to thermal expansion. The same situation was observed in the case of a Ti–4wt.% Co alloy subjected to the HPT process [40].

HPT of pure titanium also leads to ω → α transformation. However, the HPT-induced ω-phase volume fraction reached only about 40% [34]. Moreover, the reverse ω → α transitions finished by 180 °C at the heating rate of 10 °C/min [3]. Therefore, alloying with 3wt.% of Nb resulted in a twofold increase of the ω-phase volume fraction and an increase its thermal stability up to 250 °C. HPT of the Ti–4wt.% Co alloy led to the formation of the ω-phase in the amount of 65–80%, depending on the temperature of the preliminary annealing. Thermal stability of the cobalt-doped ω-phase was reached at around 450 °C [40]. In Ti–Fe alloys with iron content of 1, 2, 4 and 7 wt.%, HPT led to the formation of the ω-phase in amounts of 50, 80, 94 and 72%, respectively [29]. In these four Ti–Fe alloys, the ω-phase fully decomposed above ~600 °C [29]. Thus, the thermal stability of the Nb-doped ω-phase was higher than that of the pure Ti and lower than that of cobalt- or iron-doped samples. It should be noted that in Ti–Co and Ti–Fe systems characterised by negative mixing enthalpy, the ω-phase decomposed into the α- and intermetallic phases at higher temperature than in a system with positive mixing enthalpy (Ti–Nb), where only the reverse ω-to-α-phase transformation was observed.

**Figure 11:**
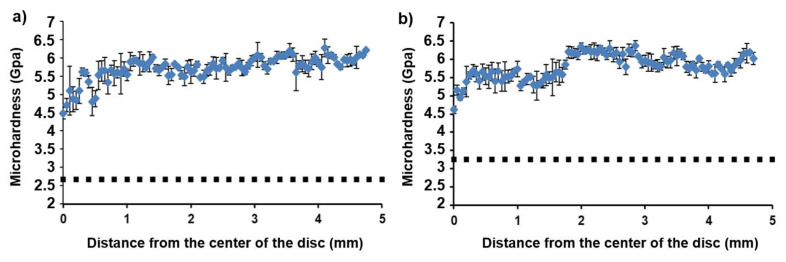
The dependence of the microhardness of the HPT-treated Ti–3wt.%Nb alloy pre-annealed at 450 °C (**a**) and 600 °C (**b**) on the distance from the disc centre. The dotted lines show the hardness in the initial states.

The measurements of the microhardness for the samples annealed at 450 and 600 °C provided values of 2.7 ± 0.4, and 3.2 ± 0.4 GPa, respectively. Figure 11 shows the dependence of microhardness after HPT on the distance from the disc centre. We observed a monotonous increase of microhardness with increasing distance from the disc centre. Such behaviour is typical for samples after HPT. At a distance of 1.5–2 mm from the centre, the hardness reached saturation for both samples. The hardness curves of the deformed samples almost coincide with each other, and their average values reach 5.7–5.8 GPa. However, the hardness increment in the sample pre-annealed at 450 °C is higher due to the presence of a higher content of the solid ω-phase in it.

The application of high pressure to Ti-based alloys (both with and without strain) permits the production of the high-pressure ω-phase. Though the ω-phase is metastable at ambient pressure, it can remain after pressure release and, therefore, can be used for tailoring the properties of Ti-based alloys. The ω-phase is fairly brittle compared with the α-phase. This issue may significantly limit the use of Ti in high-pressure conditions. However, the short annealing of pure Ti after HPT deformation can improve strength and ductility [41] and can lead to the reverse ω-to-α transformation [24]. Therefore, it can be supposed that this reverse ω-to-α transition can be used to improve the mechanical properties of Ti alloys.

## 4. Conclusions

HPT of Ti–Nb alloys resulted in a decrease of the grain size, partial α-to-ω transformation, and complete β → ω phase transformation. Two kinds of ω-phase with different chemical composition were obtained after HPT. The first was formed from the β-phase, enriched in Nb, and the second from the α-phase. The α → ω phase transformation depends on Nb concentration in the initial α-Ti phase. The less Nb in the α-phase, the more of the α-phase is transformed into the ω-phase.

The reverse ω → α transformation took place in the temperature range of ~200–250 °C. The thermal stability of the ω-phase was higher than that in pure Ti and lower than those in Ti–Co- and Ti–Fe-based alloys subjected to HPT.

## Figures and Tables

**Table 1:** Lattice parameters of all phases and the cell volume of the α-phase before and after HPT.

T, °C	Before HPT			After HPT		
	Lattice Parameters, nm		Cell Volume, nm^3^	Lattice Parameters, nm		Cell Volume, nm^3^
	α-Ti	β-Ti	α-Ti	α-Ti	ω-Ti	α-Ti
450 °C	a: 0.29552 c: 0.46941	0.32507	0.10453	a: 0.2958; c: 0.4689	a: 0.4627 c: 0.2828	0.10463
600 °C	a: 0.29514 c: 0.46912	0.32436	0.10413	a: 0.2958; c: 4.6861	a: 0.4627 c: 0.2831	0.10452
Pure Ti [34]	a: 0.29551 c: 0.46942	-	0.10452	a: 0.2959 c: 0.4690	-	0.10472

**Table 2:** Volume fraction of some phases before and after HPT as well as Nb content in the α-phase in the initial state.

T, °C	Before HPT, (α + β) State		After HPT, (α + ω) State	
	Volume Fraction of the β-Phase, %	Nb Content in the α-Phase, wt.%	Volume Fraction of the ω-Phase, %	Volume Fraction of the α-Phase, Transformed to the ω-Phase, %
450	about 4	2.2	84	80
600	about 8	3.2	79	71

## Data Availability

The data presented in this study are available on request from the corresponding author.
